# Species‐Specific Responses of Farmland Birds to Overhead Powerlines

**DOI:** 10.1002/ece3.71984

**Published:** 2025-08-15

**Authors:** Ana Teresa Marques, João Paulo Silva, Francisco Moreira

**Affiliations:** ^1^ CIBIO, Centro de Investigação em Biodiversidade e Recursos Genéticos, InBIO Laboratório Associado Universidade do Porto Vairão Portugal; ^2^ CIBIO, Centro de Investigação em Biodiversidade e Recursos Genéticos, InBIO Laboratório Associado, Instituto Superior de Agronomia Universidade de Lisboa Lisboa Portugal; ^3^ BIOPOLIS Program in Genomics, Biodiversity and Land Planning CIBIO Vairão Portugal; ^4^ Estação Biológica de Mértola (EBM) Mértola Portugal

**Keywords:** avoidance, displacement, grassland birds, impact‐gradient design, infrastructures, *Melanocorypha calandra*, *Tetrax tetrax*

## Abstract

Displacement and attraction effects caused by transmission powerlines on bird species have been documented, but this topic remains underexplored in the scientific literature. Here, we evaluated the impact of transmission powerlines on bird species typical of open farmlands in the Mediterranean region, focusing on five areas designated to protect farmland birds in Southern Portugal. Using an impact‐gradient design, we assessed how birds responded to the proximity of transmission powerlines. Breeding bird data were collected in 2021 and 2022. Our analysis revealed that, after controlling for habitat effects, overall Bird Species Richness and Grassland Bird Species Richness were not significantly influenced by the distance to powerlines. However, species‐specific negative effects were observed for two farmland species: the little bustard ( *Tetrax tetrax* ) and the calandra lark ( *Melanocorypha calandra* ), both showing displacement up to 1 km from the powerlines. These findings indicate no evidence of community‐level effects on species richness and no evidence of attraction to powerlines from farmland birds, but confirm species‐specific displacement effects, with some species negatively impacted by the presence of the transmission powerlines. The little bustard, a species of high conservation concern that is also highly susceptible to collision with overhead powerlines, requires compensation for habitat loss due to the avoidance effect caused by powerlines.

## Graphical abstract

We assessed the displacement effects of powerlines on farmland breeding birds. No significant effects were detected on the overall bird breeding community. Species‐specific negative impacts were identified for two species. Our findings underscore the need for targeted conservation strategies for powerlines.

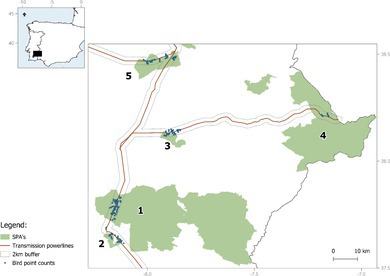

## 1 Introduction

The electric grid has been expanding over the past decades to meet growing global energy demands, a trend currently accentuated by the renewable energy transition. The need to convert to renewable energy is pushing significant investments to expand and modernize the electric network, including transmission and distribution overhead powerline grids, which are projected to expand by approximately 2 million km annually from 2022 to 2030 (IEA 2023). Like other infrastructures, the construction and operation of powerlines increase the human footprint, directly affecting biodiversity. Bird mortality due to collision or electrocution is the most reported and studied impact, but effects on other taxa, such as mammals and plants, have also been documented (Biasotto and Kindel 2018; Richardson et al. 2017).

Effects of powerlines on birds' space use, such as attraction or displacement, have been far less studied than mortality (D'Amico et al. 2018). Positive effects have been linked to the habitat heterogeneity caused by powerlines. This occurs when the vegetation along the powerline rights‐of‐way (RoW), the area beneath the overhead cables, is managed differently from its surrounding area due to powerline operational factors (Biasotto and Kindel 2018). For instance, powerlines bisecting forested landscapes may increase overall species richness and abundance by creating open or scrub habitats, thereby attracting nonforest species to the RoW (Hrouda and Brlík 2021; King et al. 2009). Similarly, in intensive farmland areas, the presence of shrubs and small trees in the RoW and below the pylons was also associated with higher bird species richness and abundance (Tryjanowski et al. 2014). However, these seemingly positive effects appear to be species‐specific, as not all species respond equally to the corridors provided by the RoW (King et al. 2009), and negative impacts are expected in species sensitive to habitat fragmentation and edge effects (King et al. 2009; Rich et al. 1994).

Powerlines can also significantly influence birds' space use through their physical structures. Electric pylons may serve as hunting and roosting perches, as well as nesting platforms, while wires are frequently used as perching sites (Biasotto and Kindel 2018; D'Amico et al. 2018). However, by providing a wide field of view and enhancing predation efficiency, pylons may attract predators such as raptors and crows (DeGregorio et al. 2014), thereby increasing predation risk for prey species.

Displacement effects have been observed even in the absence of habitat alterations within the RoW, suggesting that behavioral avoidance of the powerline structures themselves can influence species' distribution patterns. Such effects have been particularly noted in studies focusing on individual species, where structural presence alone appears sufficient to reduce habitat use near powerlines. Species specialized in open habitats appear to be particularly sensitive, with displacement caused by powerlines observed in bird species with varying life histories and ranges. Examples include the greater ( *Tympanuchus cupido* ) and lesser ( *T. pallidicinctus* ) prairie‐chickens in the Great Plains of North America (Pruett et al. 2009), the great ( *Otis tarda* ) and the little ( *Tetrax tetrax* ) bustard breeding in the farmlands of Iberia (Lane et al. 2001; Silva, Santos, et al. 2010), and breeding waders in Icelandic lowlands, particularly the Eurasian whimbrel ( *Numenius phaeopus* ) and common redshank (*Tringa tetanus*) (Pálsdóttir et al. 2022). In all these cases, birds avoid the areas closest to these structures even when there is highly suitable habitat for the species. The mechanisms underlying avoidance behavior are not fully understood, but predation risk is pointed out as a major driver, particularly due to the use of pylons by avian predators (Walters et al. 2014).

In this study, we aim to evaluate the effect of transmission powerlines on the distribution of ground‐nesting bird species typical of open farmlands in the Mediterranean region. We focus on ground‐nesting farmland birds because species from open landscapes appear particularly vulnerable to displacement caused by powerlines. This concern is supported by a previous study that reported a displacement effect on the little bustard (Silva, Santos, et al. 2010), a characteristic species of Mediterranean farmlands. Farmland birds have been experiencing significant declines over recent decades, primarily due to agricultural intensification, habitat loss, and changes in land use (Bowler et al. 2019; Gregory et al. 2019; Rigal et al. 2023). Using an impact‐gradient design, we established sampling points at varying distances from the transmission powerlines to test the hypothesis that, after controlling for habitat type, bird species richness and species presence decrease at the shortest distances from the powerlines, indicating a displacement effect on open farmland birds. While species‐specific responses to the presence of powerlines are expected, species richness was included as a complementary metric to capture broader, community‐level patterns of displacement.

## 2 Methods

### 2.1 Study Area

**FIGURE 1:**
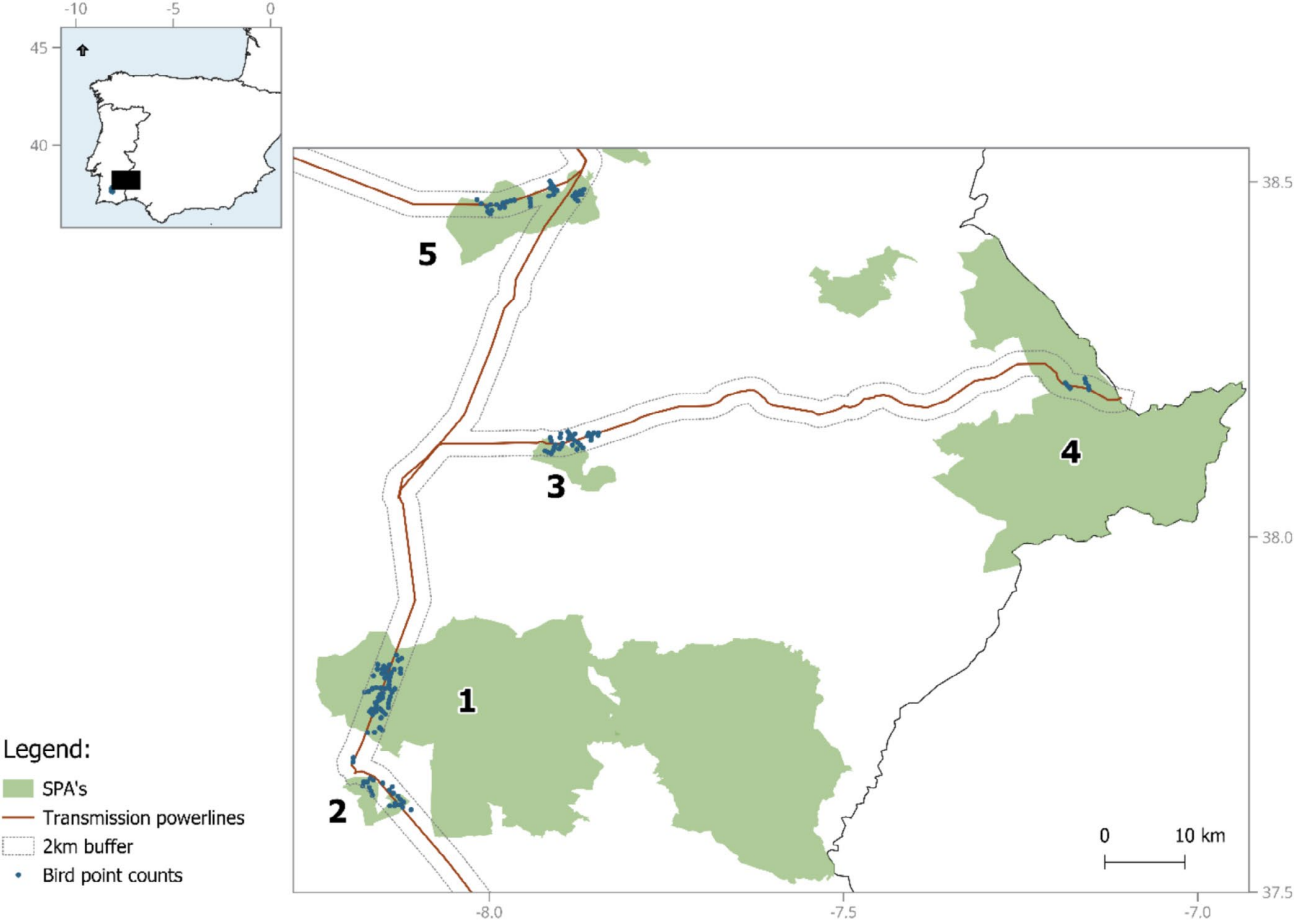
Location of the study areas, powerlines, and bird point counts. Study areas (number of sampling points): 1—Castro Verde (57); 2—Piçarras (20); 3—Cuba (32); 4—Mentiras (7, Mourão/Moura/Barrancos SPA); 5—Évora (34).

This study was carried out in the Alentejo peneplain landscapes from southern Portugal, within five Special Protection Areas and their neighboring areas: Castro Verde (PTZPE0046), Piçarras (PTZPE0058), Cuba (PTZPE0057), Mourão/Moura/Barrancos (area Mentiras; PTZPE0045), and Évora (PTZPE0055) (Figure 1). Land cover is dominated by agricultural fields, both open areas occupied by annual, nonpermanent crops and pastures, and permanent crops such as olive groves or vineyards. These SPAs are important breeding sites for farmland birds such as the corn bunting ( *Emberiza calandra* ) and the calandra lark ( *Melanocorypha calandra* ), including threatened grassland species like the little bustard and Montagu's harrier ( *Circus pygargus* ) (Gameiro et al. 2024; Moreira et al. 2007; Silva et al. 2023).

The SPA network is intersected by a total of 55.8 km of six transmission powerlines from 150 to 400 kV, configured either horizontally (with conductor wires displaced horizontally and wires at two levels) or vertically (with conductor wires and earth wire displaced vertically with four levels). The pylons' heights vary between 20 and 60 m. Further details on the powerline network can be found in Marques et al. (2020). Power lines cross the region without specific management of the RoW due to limited crop and vegetation development. Therefore, in all study areas, the habitat beneath powerlines and electric pylons (RoW) is also farmland, used by farmers and livestock as a continuation of the agricultural parcels (Figure S1). Additionally, a correlation analysis between habitat availability and distance to powerlines revealed low collinearity values (see Section 2.4), indicating that habitat composition does not consistently vary with distance from powerlines in our study areas. Thus, no habitat heterogeneity caused by the powerlines is likely.

### 2.2 Sampling Design

An impact‐gradient design was employed to assess how farmland bird distribution responds to transmission powerlines, focusing on estimating distance‐based threshold effects (Marques et al. 2021). A total of 150 sampling points (ranging from 7 to 57 points per study area; Figure 1) were placed within open farmland areas and at varying distances from the powerlines, extending up to 2 km (Figure S2). This maximum distance was chosen as it represents a point for which the anticipated effects from the structure are no longer expected (Benítez‐López et al. 2010). The number of sampling points per study area was variable, primarily due to differences in the extent of suitable habitat intersected by transmission powerlines within each Special Protection Area. Point locations were selected randomly, but with spatial constraints to ensure methodological consistency and reduce confounding factors. Specifically, a minimum distance of 600 m between points was established to prevent double bird counts, and no points were located within 500 m of major roads and urban settlements to minimize the influence of other anthropogenic structures (Moreira et al. 2012; Silva et al. 2023). Point placement was conducted using GIS tools prior to field surveys.

Bird assemblages and land uses were assessed within a 250 m buffer around each sampling point. This radius was chosen because it allows good detectability in open farmlands, allowing adequate identification of farmland bird species, and is the one used to survey the little bustard in the region (Moreira et al. 2012; Silva et al. 2023).

Land uses available at each point count were characterized by dividing the 250 m radius circle into eight “slice” sections. The dominant land use, representing the largest proportion of each section, was visually estimated for the following categories: (i) fallow land, (ii) plowed fields, (iii) cereal and hay fields, hereafter designated as cereal fields, (iv) dry legumes (including chickpea and pea), and (v) open *montados* (cork and holm oak woodlands). In most cases, the dominant cover was easily identified due to the large size of the agricultural parcels.

### 2.3 Bird Data

During the peak of the breeding season (from April 11 to May 4), each survey point was visited once and sampled during a single 5‐min session, conducted either in the early morning or late afternoon, depending on weather conditions and logistical constraints. Both acoustic and visual observations were recorded simultaneously during each session. All surveys were carried out by a single experienced observer to ensure consistency across sites. The 250 m search radius was pre‐mapped using high‐resolution aerial imagery and GPS to assist the observer in estimating distances. In cases of uncertainty, the observer visited the approximate location of the calling bird immediately after the survey to confirm whether it was within the 250 m radius.

The Castro Verde and Piçarras SPAs were surveyed in 2021, while the remaining SPAs were surveyed in 2022. Although sampling was distributed over 2 years, all surveys were conducted within the same phenological window, and natural interannual variability is not expected to significantly influence species' spatial relationships with power lines; the primary focus of this study.

All bird species observed or heard within each sampling area were recorded (as presence/absence), except those in flight over the sampling area and aquatic birds. Crested larks ( *Galerida cristata* ) and Thekla larks ( *G. theklae* ) were grouped at the genus level (hereafter referred to as *Galerida* larks) due to challenges in reliable identification during the sampling period.

### 2.4 Data Analysis

To assess the effects of transmission powerlines on the species assemblages and occurrence of individual bird species, the following parameters were computed for each sampling site: (i) total Bird Species Richness (number of species detected at site), (ii) Grassland Bird Species Richness (number of species specialized in open farmland habitats, based on Santana et al. 2017, detected at site) and (iii) Species Presence/Absence focusing on the eight species more commonly found (in over 20% of the sampling locations) (Table S1). Less frequent species (all with < 6% occurrence) were excluded due to insufficient data for robust analysis.

Generalized Additive Models (GAM) were used to identify the effect of habitat and distance to transmission powerlines on species richness and prevalence, which permits detecting nonlinear responses without needing a priori assumptions on the expected shape of such responses (Wood 2017; Zuur et al. 2009). This modeling approach was chosen based on the expectation that any potential effects of the powerline structures would be detectable primarily in their immediate vicinity and would dissipate with increasing distance. Bird Species Richness, Grassland Bird Species Richness, and the Presence of the most abundant species were used as response variables, totaling 10 GAM models. To address the interdependencies between species occurrence and farmland habitat, land use scores recorded at each sampling location were incorporated as a covariate in the fixed component of the GAM model. Each land‐use type (fallow land, plowed fields, cereals, legumes, and *montados*) was included as a covariate with values ranging from 0 to 8, based on the cumulative dominance of each land‐use type within the eight “slice” sections of the 250 m sampling radius.

The study area was also included as a covariate, thereby accounting for potential variation in bird assemblages because of different particularities in each SPA. We used Castro Verde SPA as a reference, as it is the most relevant SPA for this group of species in terms of total open farmland surface and bird population size.

We used the Spearman correlation coefficient and variance inflation factors to check for collinearity between the explanatory variables (Zuur et al. 2009). All VIF values were below 3.0, and all pairwise correlations had |*r*| < 0.60, indicating low collinearity among the covariates; therefore, all variables were retained in the analysis. GAMs fitted with a Poisson distribution and a log link function were used to model Bird Species Richness and Grassland Bird Species Richness, and GAMs with a Binomial distribution and a logit link were used to fit species Presence. The modeling procedure involved fitting the full model, followed by backward elimination of nonsignificant (*p* > 0.05) variables to find the optimal model (Zuur et al. 2009). The optimal smoothing parameter was estimated by restricted maximum likelihood estimation (REML), and a basis dimension (*k* = 3) was defined to allow some complexity in the functions while avoiding overfitting the data. To assess model assumptions, we tested for overdispersion by calculating the overdispersion ratio (residual deviance divided by residual degrees of freedom). Spline correlogram plots with 95% pointwise confidence intervals were generated for each study area using 1000 bootstrap resamples to assess spatial autocorrelation in model residuals (Bjørnstad and Falck 2001). The low number of sample points prevented reliable estimation of the correlogram for the Mentiras area.

GAMs were fitted using the package “mgcv” (Wood 2018) and plotted using “mgcViz” (Fasiolo et al. 2020); correlograms were estimated with the ncf package (Bjørnstad 2016) in R Statistical Software (R Core Team 2023).

## 3 Results

### 3.1 Farmland Bird Patterns

A total of 41 bird species were recorded across the 150 sampling points (Table S1). The mean Bird Species Richness per sampling location was 4.94 (±1.39 SD), with Castro Verde (5.25 ± 1.23 SD), Évora (5.06 ± 1.18 SD), and Piçarras (5.05 ± 1.91 SD) exhibiting the higher values (Table S2). The majority of the sampled species were grassland species, with Castro Verde (4.33 ± 1.06 SD) and Évora (4.12 ± 1.07 SD) demonstrating higher Grassland Bird Species Richness (Table S2).

Corn bunting was the most prevalent species (95.3% of the sites), followed by *Galerida* larks (74.0%), zitting cisticola ( *Cisticola juncidis* ) (70.7%), common quail ( *Coturnix coturnix* ) (58.0%), calandra lark (47.3%), little bustard (30.7%), stonechat ( *Saxicola rubicola* ) (30.0%), and common hoopoe ( *Upupa epops* ) (20.7%) (Table S1). Most of the recorded species fall under the category of “Least Concern” according to the 2021 European Red List of Birds, except the little bustard, great spotted cuckoo ( *Clamator glandarius* ), and Iberian gray shrike ( *Lanius meridionalis* ) that are classified as “Vulnerable,” and the black‐bellied sandgrouse ( *Pterocles orientalis* ) classified as “Endangered” (Table S1; European Commission 2022). The little bustard is also classified as “Critically Endangered” in Portugal (Almeida et al. 2022), due to a significant range contraction and declining breeding population (Silva et al. 2023).

### 3.2 Factors Influencing Bird Species Richness and Species Presence

**FIGURE 2:**
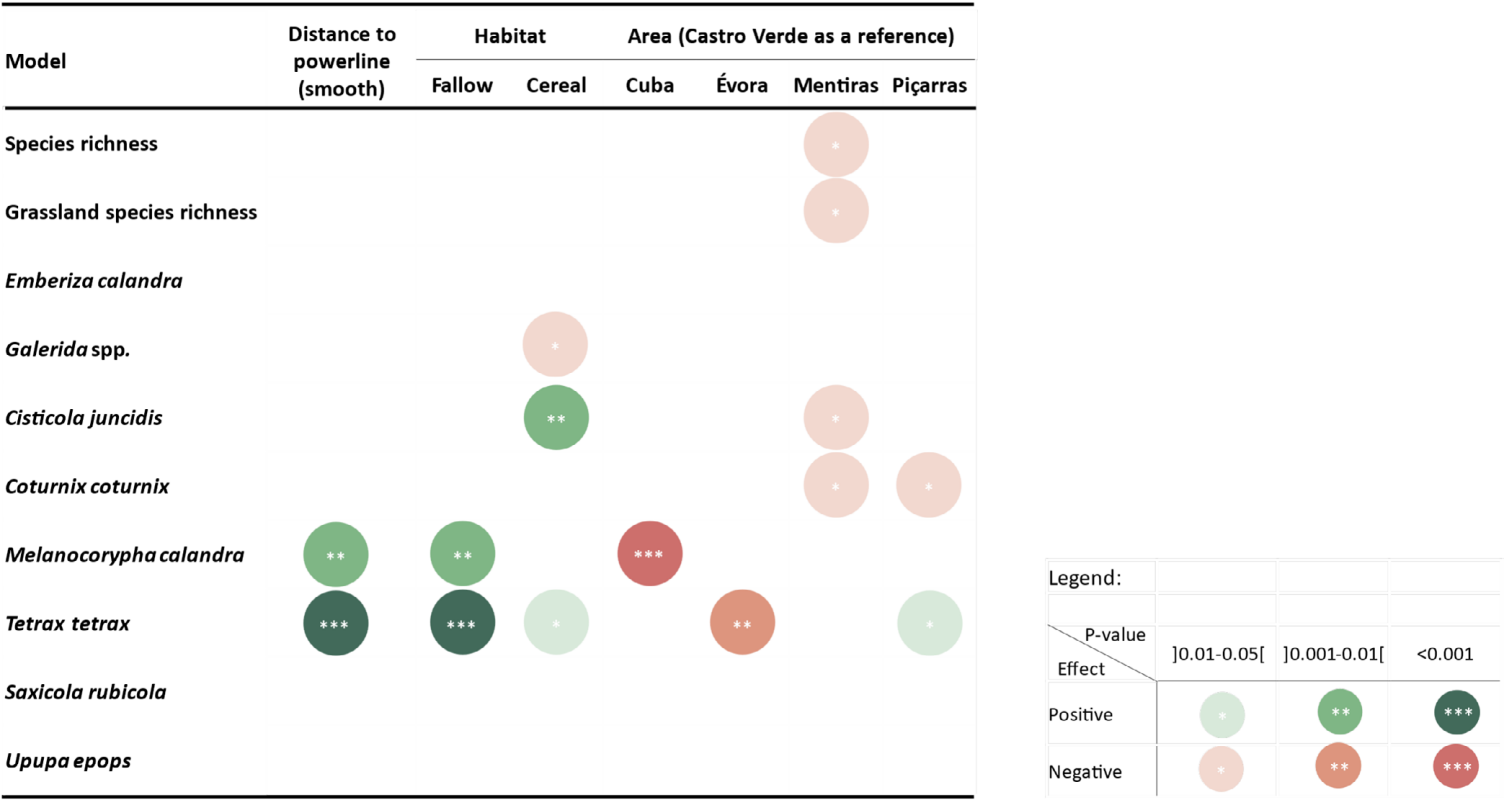
Graphical representation of the importance of the distance to powerline, habitat, and study area in explaining the bird species richness and species presence/absence near transmission powerlines in the special protection areas (SPA) within open farmlands in Alentejo, southern Portugal (April–May 2021 and 2022).

**FIGURE 3:**
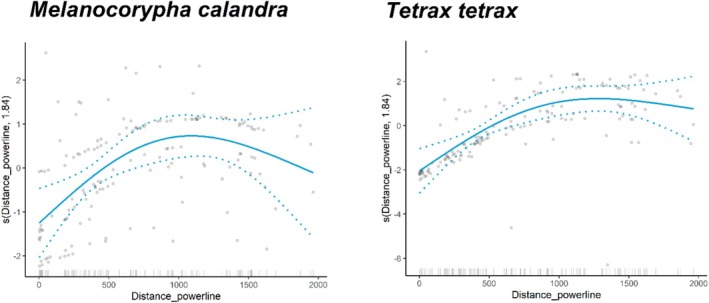
Effect of the distance to transmission powerlines on the distribution (presence/absence) of the calandra lark *Melanocorypha calandra* and little bustard *Tetrax tetrax* , fitted with GAM models. Only significant effects from powerlines are presented. Dashed lines represent 95% confidence intervals, gray dots represent the partial residual, and marks along the *x*‐axis represent a single observation. The *y*‐axis shows the contribution of the fitted centered smooth terms *s* (names of the predictor, estimated degrees of freedom) to the response variable (presence/absence of the species), after taking into account the remaining variables in the model. Summary statistics of the GAMs are provided in Table S2.

Generalized Additive Models showed that overall Bird Species Richness and Grassland Bird Species Richness were not affected by the distance to powerlines (Figure 2, Table S3). A negative effect of powerline proximity was detected on two species, the little bustard and the calandra lark (Figure 2, Table S3), with both species being affected up to 1 km distance from powerlines (Figure 3). The models for little bustard and calandra lark presence had the highest explanatory power, 32.2% and 32%, respectively, compared to 0%–21% for the other models (Table S3). No positive effects from transmission powerlines were detected.

With respect to the habitat, the little bustard was positively associated with fallow land, as well as cereal and hay fields. Similarly, the calandra lark showed a positive association with fallow land, while the zitting cisticola was positively linked to cereal and hay fields. In contrast, Galerida larks were negatively associated with cereal and hay fields (Figure 2, Table S3, Figures S3–S9). Dry legumes, plowed fields, and *montados* were not significant in any model.

Compared to Castro Verde SPA, the Mentiras study area, located in Mourão/Moura/Barrancos SPA, had a lower Species Richness value and reduced zitting cisticola and common quail presence. Little bustard presence was reduced in the Évora study area and increased in Piçarras when compared to Castro Verde. The Cuba study area had reduced presence of calandra lark (Figure 2, Table S3, Figures S3–S9).

Model diagnostics indicated, for all GAM models, no evidence of overdispersion (overdispersion ratio < 1.5) and no significant spatial autocorrelation in the model residuals (Figures S10–S16).

## 4 Discussion

In this study, we found that transmission powerlines did not have a significant impact on the overall species richness of farmland bird breeding communities, after controlling for the effects of other important covariates, such as habitat and study area. However, we identified species‐specific effects in two species, the little bustard and the calandra lark (out of a subset of eight), which exhibited lower frequencies than expected relative to the available habitat. Overall, these findings highlight the importance of considering species‐specific responses when evaluating and mitigating the effects of powerlines and other anthropogenic infrastructures.

### 4.1 Farmland Bird Distribution in Relation to Transmission Powerline Presence

In our study areas, the transmission powerlines do not negatively impact habitat availability, as the habitats of the RoW and pylons are the same as the surrounding environment (Figure S1). Therefore, the effects reported here can be specifically attributed to the structure and operation of the powerlines, rather than habitat changes associated with their presence.

Displacement or attraction effects caused by transmission powerlines did not influence local farmland bird species richness in our study areas. This finding contrasts with previous studies, which reported positive effects of powerlines at the community level, such as increased species richness, in areas where the RoW impacted habitat features, such as the creation of edges and increased landscape heterogeneity (Hrouda and Brlík 2021; King et al. 2009; Tryjanowski et al. 2014).

Both the little bustard and the calandra lark were observed less frequently in proximity to powerlines, with these negative effects extending up to 1 km from the structures. Previous studies have reported displacement effects caused by powerlines on the little bustard (Santos et al. 2016; Silva, Santos, et al. 2010), but to our knowledge, this is the first study to identify a displacement effect for a passerine species, the calandra lark. The common trait that distinguishes these two species from the others included in this study is that they are both specialists of fallow land during the breeding season, as shown in Figure 2. Additionally, both species are more abundant in large agricultural fields during the breeding season and are sensitive to habitat fragmentation effects (Morgado et al. 2010; Reino et al. 2010; Silva, Palmeirim, and Moreira 2010). This preference for more continuous habitats, combined with their avoidance of powerlines, suggests that powerline structures may contribute to habitat fragmentation. Still, these two species display different levels of vulnerability to collision with powerlines: the little bustard is highly susceptible, with documented high collision rates, whereas the mortality of calandra larks due to collisions has not been reported in the study area or in similar habitats (Barrientos et al. 2012; Marques et al. 2007).

Our findings of species‐specific effects of powerlines on farmland bird species in open habitats are consistent with the well‐known pattern that the effects of anthropogenic infrastructures such as roads, wind farms, and powerlines vary among species. While the traits of species most vulnerable to mortality from these infrastructures are generally well understood (Bernardino et al. 2018; Marques et al. 2014), less is known about other types of impacts, such as displacement. This knowledge gap is partly due to the common use of aggregated biodiversity indicators or studies focusing on a limited number of species (de Jonge et al. 2022). As a result, data on species‐specific responses to infrastructure remain scarce, hindering a comprehensive understanding of the traits shared by species most vulnerable to nonmortality impacts.

### 4.2 Other Drivers Influencing Bird Species Richness and Species Presence

The study area played an important role in explaining some of the observed species' prevalence. This outcome was expected, as although the sampling sites are located within or near SPAs, the habitat quality within these designated areas is not uniform. Overall, open farmland habitat availability varies between study areas due to differences in agricultural practices (Gameiro et al. 2020), which are often influenced by the management decisions of individual landowners and farming operations in each region. The Mentiras study area, located within the Mourão/Moura/Barrancos SPA, features the smallest and most fragmented open farmland habitat among the surveyed sites. This habitat fragmentation likely contributed to the reduced overall species richness and lower abundance of grassland species observed in the area. In the remaining areas, the main differences observed are associated with species specialized in fallow land. In the Cuba area, the occurrence of the calandra lark is lower than expected given the available habitat, while in Évora, the same pattern is observed for the little bustard. While this suggests potential differences in local conditions or management, agricultural practices were not included in our analysis; thus, we cannot draw conclusions regarding their role. It is worth noting, however, that Castro Verde benefits from a long‐standing agri‐environmental scheme, which, along with its extensive open farmland, may contribute to its higher conservation value.

It is important to note that the number of sampling points varied across SPAs due to differences in the extent of suitable habitat intersected by transmission powerlines. This resulted in an unbalanced sampling design; however, it reflects the distribution of both infrastructure and habitat in the region. Still, this variation may have influenced some of the observed patterns in species prevalence. This is particularly relevant in the Mentiras study area, which had a significantly lower number of sampling points (*n* = 7) due to limited overlap between powerlines and open farmland. Notably, this area also recorded the lowest species richness among all studied SPAs.

Habitat characteristics were also important in explaining species prevalence across the study areas, a pattern consistent with findings from previous studies conducted in the region. As expected, the calandra lark and the little bustard were strongly associated with fallow land, while the zitting cisticola was linked to cereal fields, reflecting established habitat preferences (Delgado and Moreira 2000). The positive association of the little bustard with cereal and hay fields, though weaker than its preference for fallow land, was somewhat unexpected. This finding may be partly explained by the observed limited vegetative development of most cereal fields (therefore making them structurally similar to fallow land) during the studied springs, likely driven by climatic factors.

### 4.3 Management Implications

Like other members of the Otididae family, little bustards are highly vulnerable to collisions with powerlines. Although mitigation measures such as wire‐marker devices may reduce collision events (Marques et al. 2020), they are insufficient to fully address the impacts of these structures (Silva et al. 2022). The displacement effect on little bustards identified in this study further highlights the negative impacts of powerlines and underscores the importance of avoiding the construction of overhead powerlines in areas where this species occurs. Burying powerlines or routing them away from key areas for the species will effectively prevent both mortality and displacement of the species. These mitigation measures are especially crucial given the species' severe population decline in Western Europe (Morales and Bretagnolle 2021) and its high sensitivity to such infrastructure.

It is also worth noting that existing transmission powerlines are significantly impacting the breeding habitat of the little bustard. A rough estimation of the species' potential breeding habitat (areas with annual crops and pastures, as identified in Portugal's 2018 Land Cover Map; DGT 2022) affected by the displacement effect, defined as the potential habitat within a 1 km buffer of powerlines crossing SPAs where the species breeds, indicates that approximately 6320 ha of potential habitat within the Alentejo SPAs are impacted. Displacement cannot be fully mitigated unless powerlines are decommissioned and re‐routed, as is currently happening in the SPA of Castro Verde. Given that the little bustard is currently classified as Critically Endangered in Portugal (Almeida et al. 2022), compensatory measures should be considered for the existing transmission powerlines crossing SPAs with breeding populations of this species.

The observed displacement of the calandra lark from areas near transmission powerlines represents an important and novel finding. Unlike bustards, whose high collision risk has prompted mitigation measures during powerline planning and development, larks are generally not considered vulnerable to such threats and are therefore often overlooked in impact assessments. However, our results suggest that the presence of transmission powerlines may affect calandra larks through habitat avoidance, a mechanism that has received little attention in conservation planning. Although the species is currently listed as Least Concern globally and in Europe, it has recently been classified as Near Threatened in Portugal (Almeida et al. 2022), reflecting regional declines. This highlights the need to re‐evaluate the potential effects of infrastructure beyond collision risk alone. Conservation and mitigation strategies should therefore incorporate broader assessments of infrastructure impacts, particularly in areas of high importance for declining farmland bird populations such as the calandra lark.

The exclusion effects caused by infrastructure such as powerlines are still relatively poorly understood, and there is a limited number of studies that specifically address this issue. Gaining a deeper understanding of this phenomenon is crucial for identifying which species are most affected and what species‐specific traits contribute to their sensitivity. Further research in this area is essential to uncover the mechanisms driving avoidance behavior and to inform more effective conservation and mitigation strategies.

To effectively mitigate infrastructure‐related impacts, conservation strategies must go beyond general habitat protection and consider the specific sensitivities of affected species. In areas of high conservation value, such as open farmland within the breeding range of bustards, this may require targeted interventions, including the rerouting or undergrounding of powerlines. Advancing our understanding of species responses to infrastructure is essential for developing more informed and effective mitigation measures.

## Author Contributions

**Ana Teresa Marques:** conceptualization (equal), data curation (lead), formal analysis (lead), investigation (equal), methodology (equal), writing – original draft (lead). **João Paulo Silva:** conceptualization (equal), methodology (equal), writing – review and editing (equal). **Francisco Moreira:** conceptualization (equal), methodology (equal), writing – review and editing (equal).

## Conflicts of Interest

The authors declare no conflicts of interest.

## Supporting information


**Appendix S1:** ece371984‐sup‐0001‐AppendixS1.pdf.

## Data Availability

The dataset used in this study is publicly available in Dyrad Digital Repository: https://datadryad.org/dataset/doi:10.5061/dryad.np5hqc06d.
