# Supplementary material for: Species‐Specific Responses of Farmland Birds to Overhead Powerlines
Source: Ecol Evol. 2025 Aug 15;15(8):e71984. doi: 10.1002/ece3.71984 (PMC12356646; doi:10.1002/ece3.71984)
Supplement: Supplementary file 1 — Appendix S1: ece371984‐sup‐0001‐AppendixS1.pdf. [file ECE3-15-e71984-s001.pdf]

Species-specific responses of farmland birds to overhead powerlines

Ana Teresa Marques, João Paulo Silva, Francisco Moreira

Ecology and Evolution, 2025

Supplementary Material

TABLES

Table S1 – List of breeding bird species recorded near transmission powerlines in the Special Protection Areas (SPA) within open farmlands in Alentejo, southern Portugal (April-May 2021 and 2022). The European Red List of Birds (BirdLife International, 2021) and the Frequency of occurrence (percentage of sampling sites where the species was recorded; n=150) are presented for each species. Species underlined were taken into account in the species level analysis and species considered as typical from grasslands are highlighted with an "x" (Grassland).

Scientific name	Common name	European Red List Category	Frequency of occurrence	Grassland
Emberiza calandra	Corn Bunting	LC	95.3	x
*Galerida theklae/G. cristata**	Thekla lark/ Crested lark	LC/ LC	74.0	x
Cisticola juncidis	Zitting Cisticola	LC	70.7	x
Coturnix coturnix	Common Quail	NT	58.0	x
Melanocorypha calandra	Calandra Lark	LC	47.3	x
Tetrax tetrax	Little Bustard	VU	30.7	x
Saxicola rubicola	Common Stonechat	LC	30.0	
Upupa epops	Common Hoopoe	LC	20.7	
Alectoris rufa	Red-legged Partridge	NT	6.0	
Carduelis carduelis	European Goldfinch	LC	6.0	
Passer hispaniolensis	Spanish Sparrow	LC	5.3	
Lullula arborea	Woodlark	LC	4.7	
Circus pygargus	Montagu's Harrier	LC	4.0	x
Lanius senator	Woodchat Shrike	NT	3.3	
Burhinus oedicnemus	Eurasian Thick-knee	LC	3.3	x
Calandrella brachydactyla	Greater Short-toed Lark	LC	3.3	x
Clamator glandarius	Great Spotted Cuckoo	VU	2.7	
Pterocles orientalis	Black-bellied Sandgrouse	EN	2.7	x
Turdus merula	Eurasian Blackbird	LC	2.7	
Parus major	Great Tit	LC	2.0	
Sturnus unicolor	Spotless Starling	LC	2.0	
Athene noctula	Little owl	LC	2.0	

Scientific name	Common name	European Red List Category	Frequency of occurrence	Grassland
Certhia brachydactyla	Short-toed Treecreeper	LC	1.3	
Columba palumbus	Common Woodpigeon	LC	1.3	
Corvus corone	Carrion Crow	LC	1.3	
Fringilla coelebs	Common Chaffinch	LC	1.3	
Lanius meridionalis	Iberian Grey Shrike	VU	1.3	
Streptopelia decaocto	Eurasian Collared-dove	LC	1.3	
Otis tarda	Great Bustard	LC	0.7	x
Anthus campestris	Tawny Pipit	LC	0.7	x
Ciconia ciconia	White Stork	LC	0.7	
Cuculus canorus	Common Cuckoo	LC	0.7	
Falco naumanni	Lesser Kestrel	LC	0.7	x
Falco tinnunculus	Common Kestrel	LC	0.7	
Buteo buteo	Eurasian Buzzard	LC	0.7	
Elanus caraeruleus	Black-winged Kite	LC	0.7	
Hieraetus pennatus	Booted Eagle	LC	0.7	
Hirundo rustica	Barn Swallow	LC	0.7	
Linaria cannabina	Common Linnet	LC	0.7	
Merops apiaster	European Bee-eater	LC	0.7	
Passer domesticus	House Sparrow	LC	0.7	
Passer montanus	Eurasian Tree Sparrow	LC	0.7	

* Due to difficulty in accurately identifying all individuals of these two species in the field, the Crested and Thekla larks (Galerida cristata and G. theklae) were categorized to the genus level.

Table S2 – Bird Species Richness and Grassland Bird Species Richness (mean number of species per sampling site ± standard deviation) per study area.

Area	Bird Species Richness	Grassland Bird Species Richness
1 - Castro Verde	5.25±1.23	4.33±1.06
2 - Piçarras	5.05±1.91	3.33±1.46
3 - Cuba	4.38±1.21	3.53±1.16
4 - Mentiras	3.43±1.13	2.43±1.13
5 - Évora	5.06±1.18	4.12±1.07
All areas	4.94±1.39	3.89±1.24

Table S3 – Summary statistics for the ten GAM models (Bird Species Richness and Species Presence/ Absence). SE: standard error; z value: Wald statistic; edf: estimated degrees of freedom.

Model	Model coefficients	Estimate	SE	z value	edf	p-value	Deviance explained (%)
Bird Species Richness	**Intercept**	**1.66**	**0.06**	**28.45**		**0.000**	10.5
	Area (Castro Verde as a reference)						
	Cuba	-0.18	0.10	-1.77		0.08	
	Évora	-0.04	0.10	-0.38		0.70	
	**Mentiras**	**-0.42**	**0.21**	**-2.00**		**0.045**	
	Piçarras	-0.04	0.11	-0.34		0.73	
Grassland Bird Species Richness	**Intercept**	**1.47**	**0.06**	**23.05**		**0.000**	16.1
	Area (Castro Verde as a reference)						
	Cuba	-0.20	0.11	-1.80		0.07	
	Évora	-0.05	0.11	-0.48		0.63	
	**Mentiras**	**-0.58**	**0.25**	**-2.31**		**0.02**	
	Piçarras	-0.26	0.13	-1.94		0.05	
Emberiza calandra	Without significant covariates						
Galerida spp.	**Intercept**	**1.07**	**0.19**	**5.57**		**0.000**	4.34
	**s(Cereal)**				**1.24**	**0.02**	
Cisticola juncidis	**Intercept**	**1.47**	**0.40**	**3.69**		**0.000**	21
	**s(Cereal)**				**1**	**0.005**	
	Area (Castro Verde as a reference)						
	Cuba	0.54	0.65	0.82		0.41	
	Évora	0.06	0.52	0.11		0.91	
	**Mentiras**	**-3.07**	**1.26**	**-2.43**		**0.02**	
	Piçarras	-0.99	0.56	-1.78		0.08	
Coturnix coturnix	**Intercept**	**0.54**	**0.27**	**1.96**		**0.05**	6.48
	Area (Castro Verde as a reference)						
	Cuba	0.25	0.47	0.53		0.60	
	Évora	-0.06	0.45	-0.13		0.90	
	**Mentiras**	**-2.33**	**1.11**	**-2.09**		**0.04**	
	**Piçarras**	**-1.23**	**0.54**	**-2.29**		**0.02**	
Melanocorypha calandra	Intercept	0.37	0.31	1.17		0.24	32
	**s(Distance to powerline)**				**1.84**	**0.015**	
	**s(Fallow)**				**1.73**	**0.005**	
	Area (Castro Verde as a reference)						
	**Cuba**	**-2.69**	**0.72**	**-3.73**		**0.000**	
	Évora	0.20	0.53	0.37		0.71	
	Mentiras	-1.64	1.16	-1.41		0.16	
	Piçarras	-0.66	0.64	-1.03		0.30	
Tetrax tetrax	**Intercept**	**-1.12**	**0.43**	**-2.63**		**0.009**	32.2
	**s(Distance to powerline)**				**1.84**	**0.000**	

Model	Model coefficients	Estimate	SE	z value	edf	p-value	Deviance explained (%)
	**s(Fallow)**					**0.000**	
	**s(Cereal)**					**0.011**	
	Area (Castro Verde as a reference)						
	Cuba	-1.11	0.67	-1.65		0.10	
	**Évora**	**-1.58**	**0.58**	**-2.71**		**0.007**	
	Mentiras	0.15	1.28	-0.12		0.91	
	**Piçarras**	**2.67**	**0.16**	**2.21**		**0.03**	
Saxicola torquata	Without significant covariates						
Upupa epops	Without significant covariates						

FIGURES

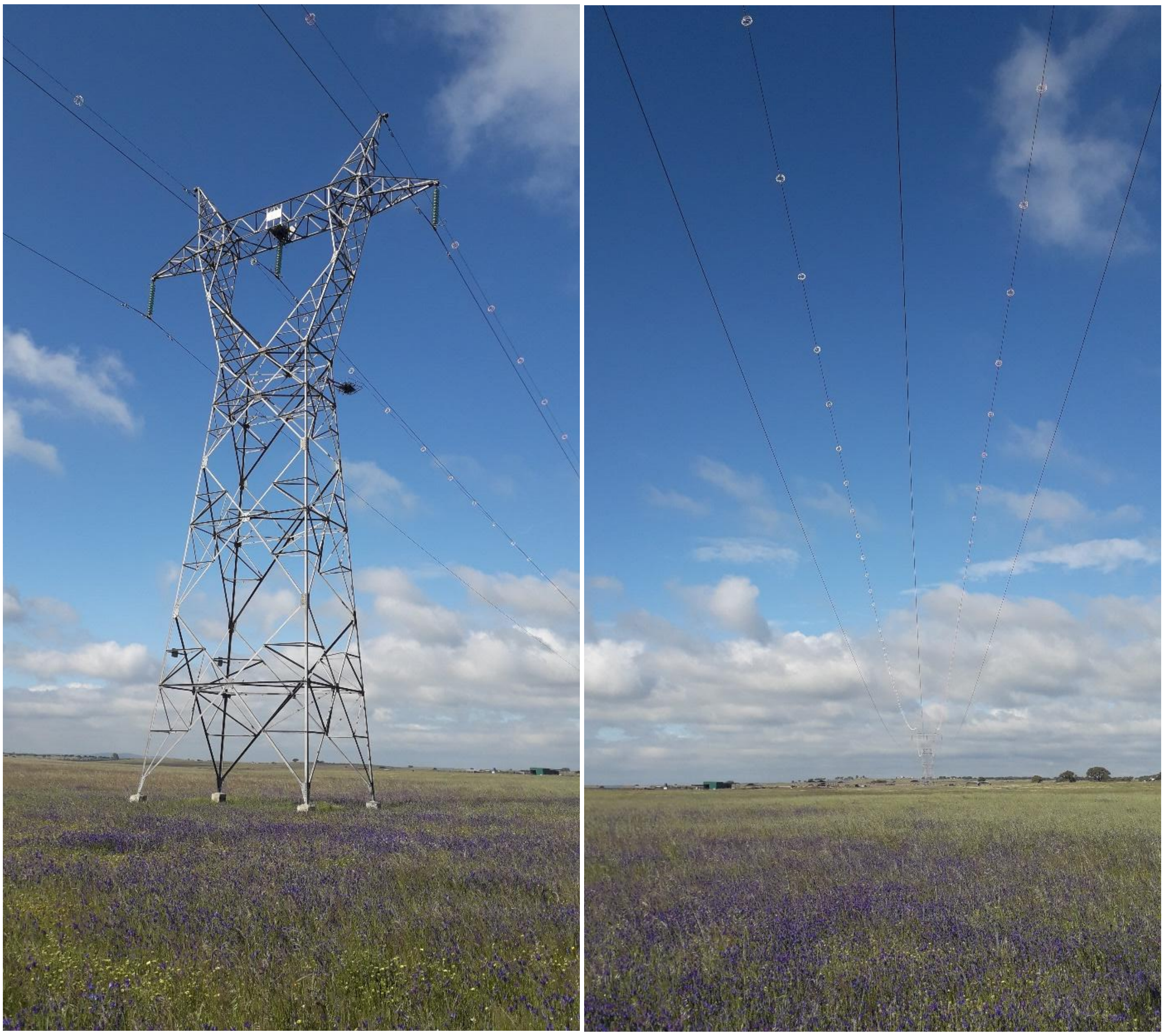

Figure S1 – Example of a transmission powerline in an open farmland habitat of Alentejo, Portugal.

Note the continuous habitat beneath the pylon and along the rights-of-way (RoW) corridor.

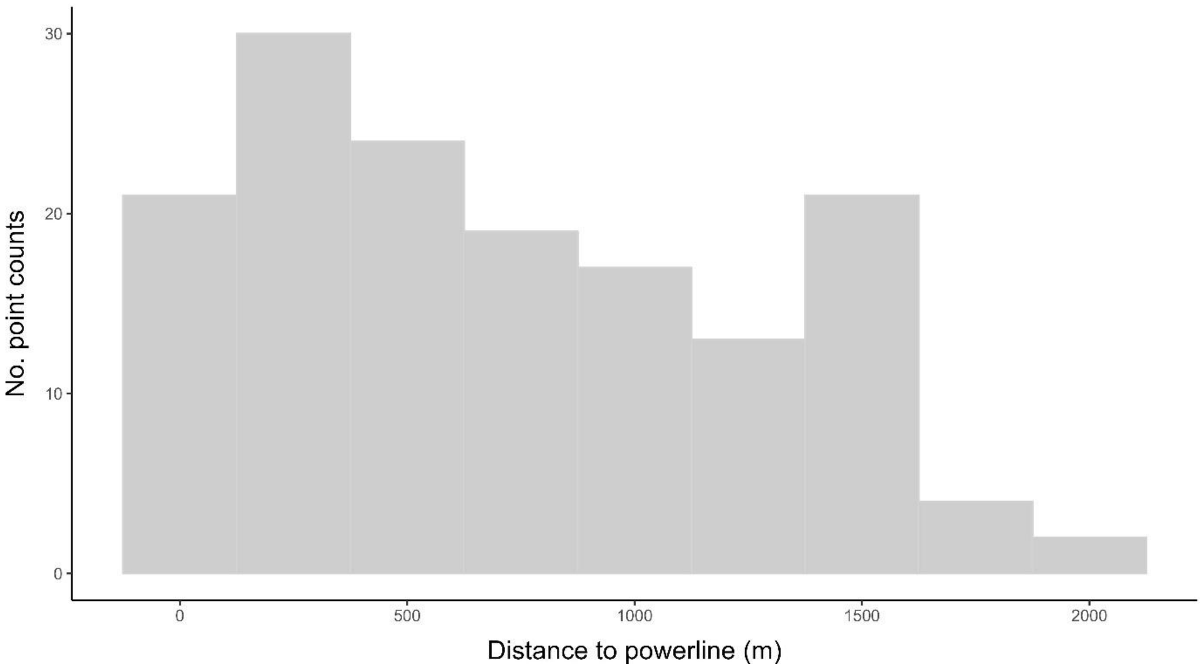

Figure S2 – Distribution of sampling point counts in relation to the distance to a powerline (m).

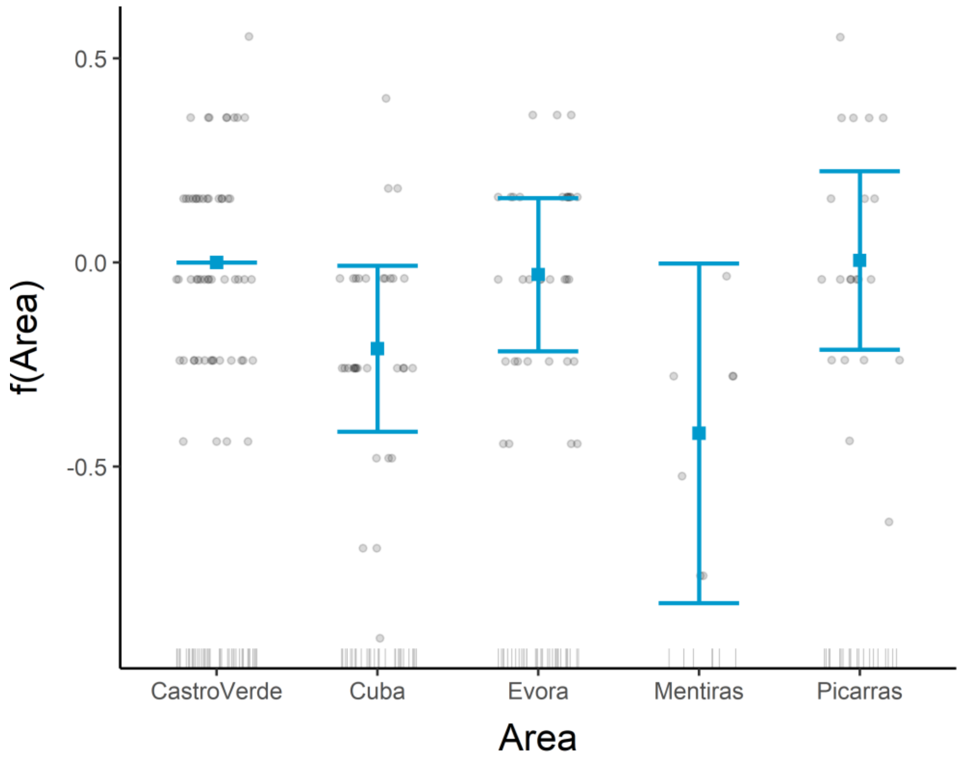

Figure S3 – Effect of the study area on the **Bird Species Richness**. Grey dots represent the partial residual, and marks along the x-axis represent a single observation. Summary statistics of the GAM model is provided in Table S3.

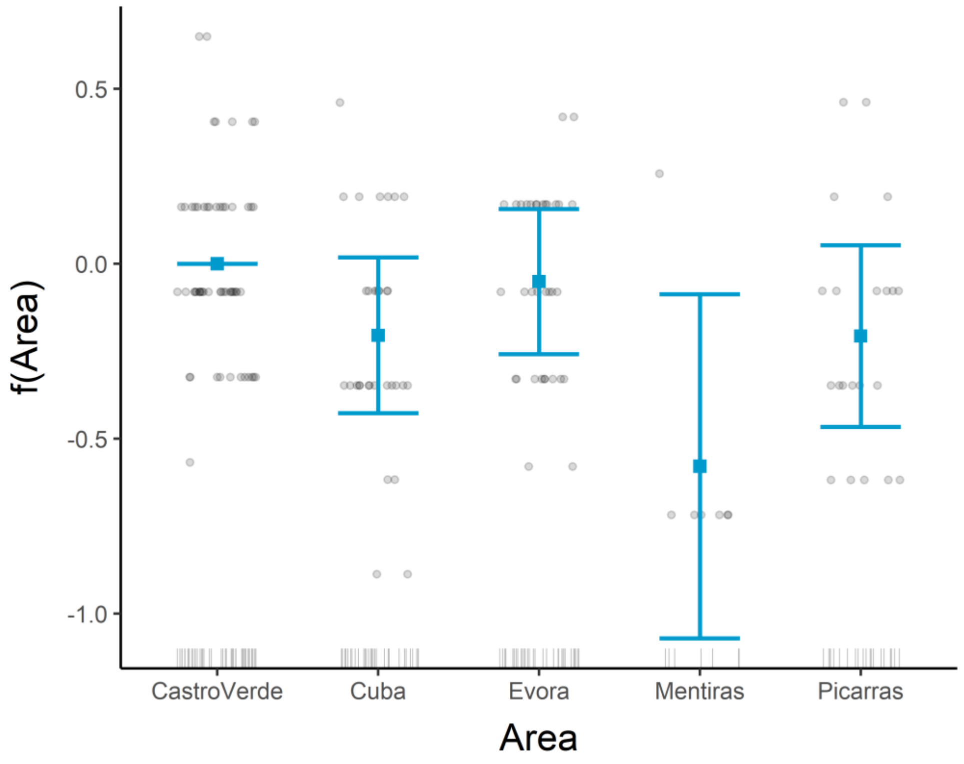

Figure S4 – Effect of the study area on the **Grassland Bird Species Richness**. Grey dots represent the partial residual, and marks along the x-axis represent a single observation. Summary statistics of the GAM model is provided in Table S3.

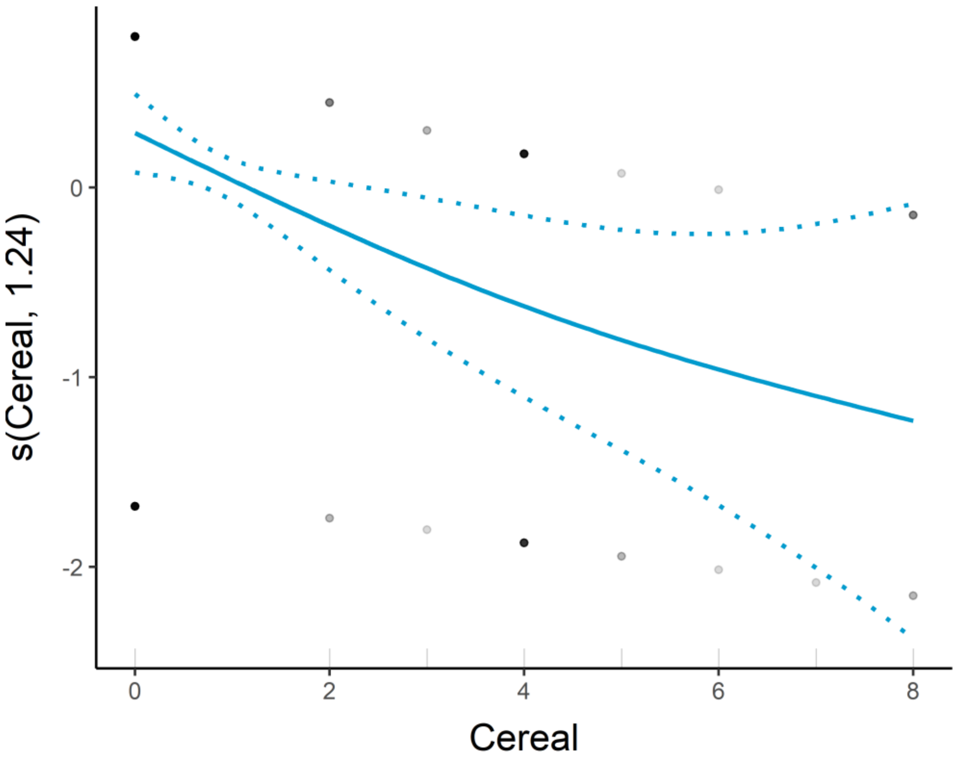

Figure S5 – Effect of cereal abundance on the distribution (presence/ absence) of the **galerida larks *Galerida* spp.**. Dashed-lines represent 95% confidence intervals, grey dots represent the partial residual, and marks along the x-axis represent a single observation. Summary statistics of the GAM model is provided in Table S3.

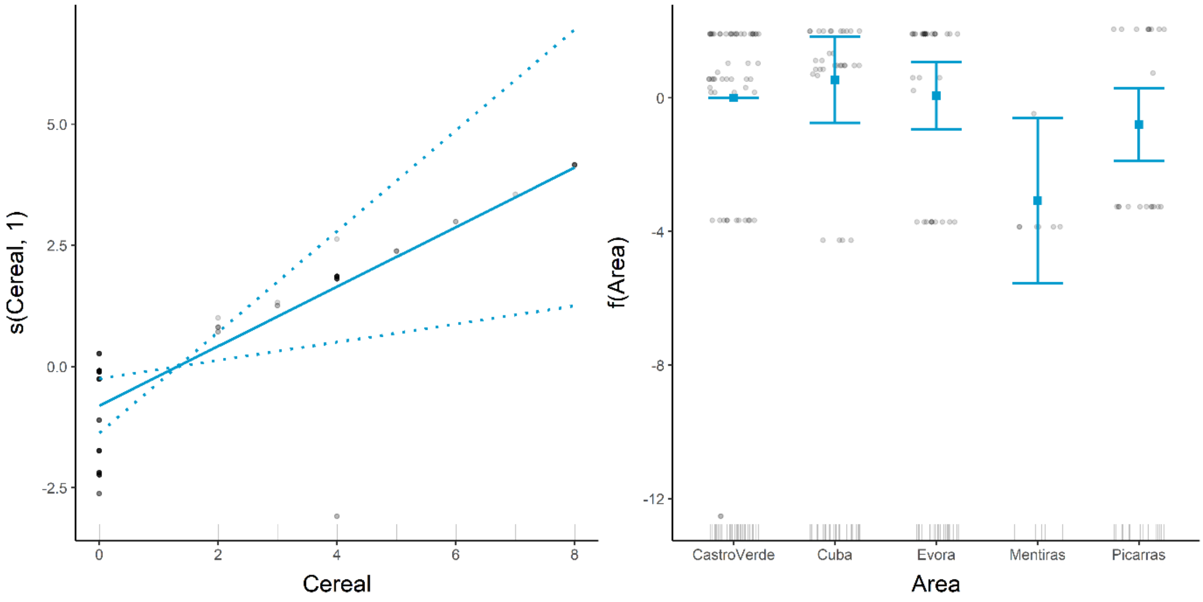

Figure S6 – Effect of the study area and cereal abundance on the distribution (presence/ absence) of the **zitting cisticola *Cisticola juncidis***. Dashed-lines represent 95% confidence intervals, grey dots represent the partial residual, and marks along the x-axis represent a single observation. Summary statistics of the GAM model is provided in Table S3.

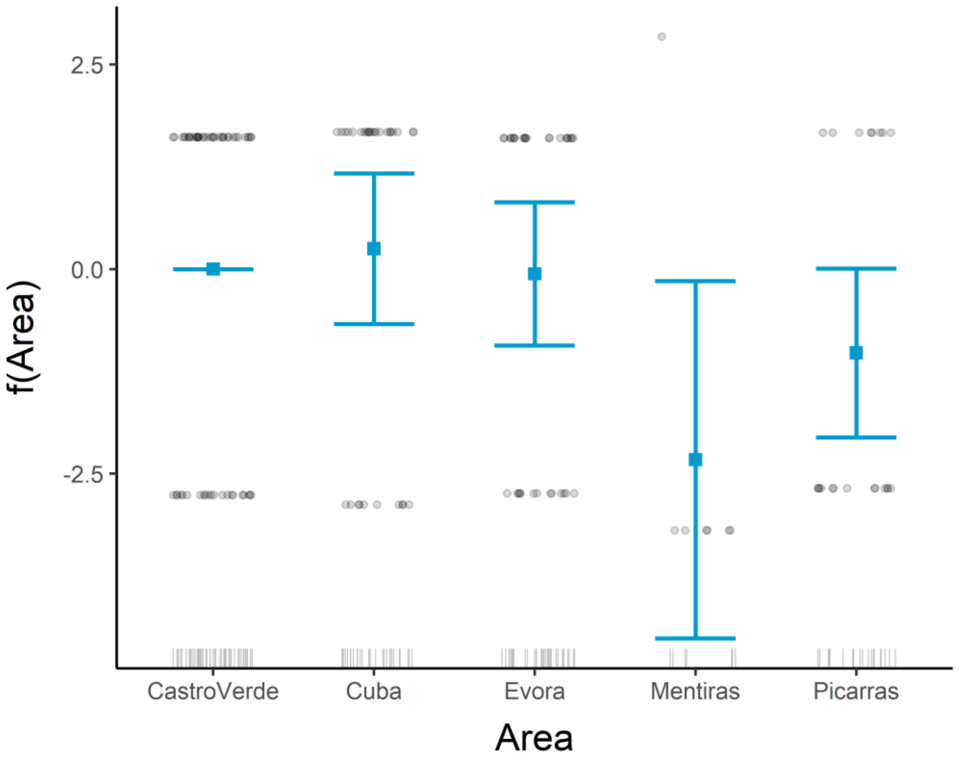

Figure S7 – Effect of the study area on the **common quail *Coturnix coturnix***. Grey dots represent the partial residual, and marks along the x-axis represent a single observation. Summary statistics of the GAM model is provided in Table S3.

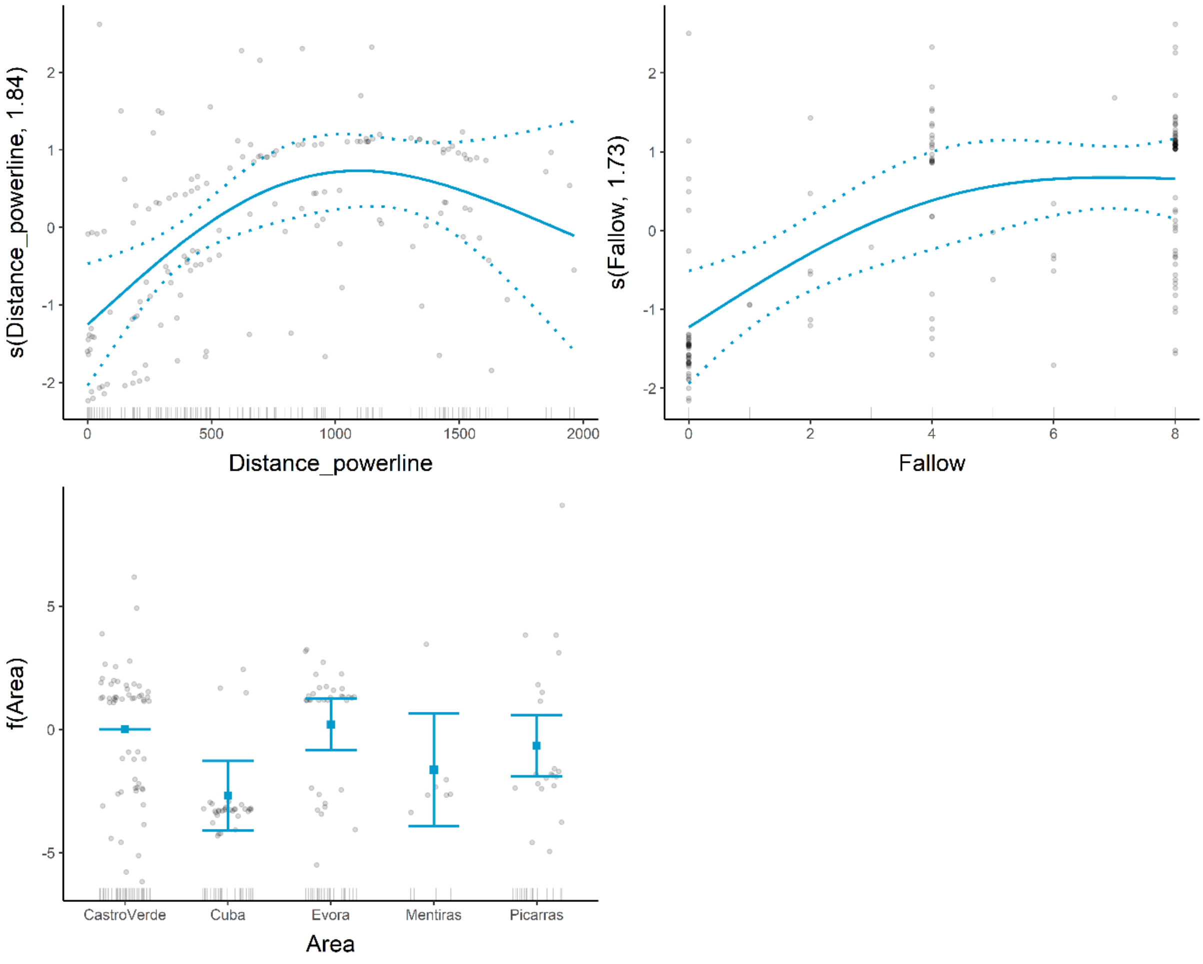

Figure S8 – Effect of the distance to transmission powerlines, study area and fallow land abundance on the distribution (presence/ absence) of the **calandra lark *Melanocorypha calandra***. Dashed-lines represent 95% confidence intervals, grey dots represent the partial residual, and marks along the x-axis represent a single observation. Summary statistics of the GAM model is provided in Table S3.

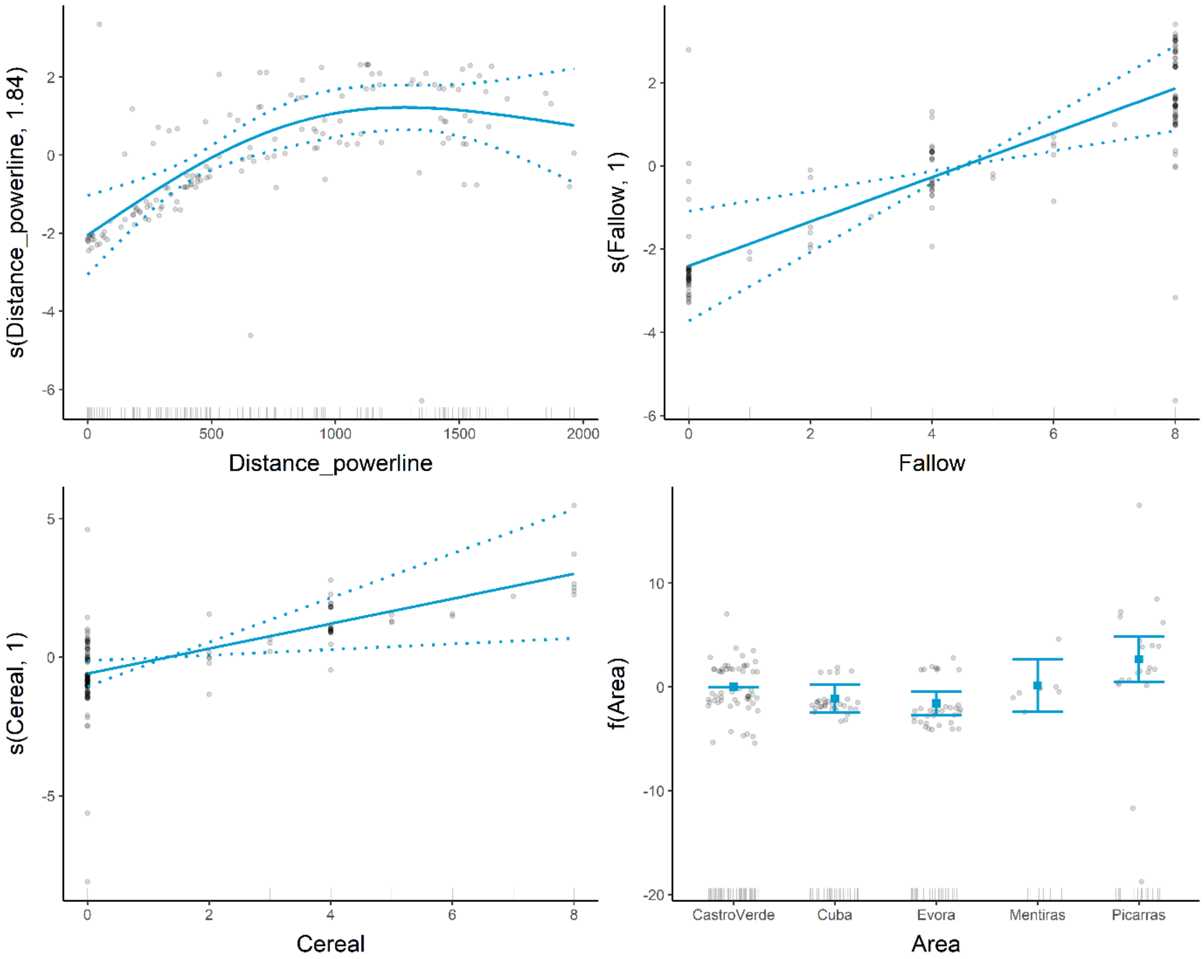

Figure S9 – Effect of the distance to transmission powerlines, study area, fallow land abundance, and cereal abundance on the distribution (presence/ absence) of the **little bustard *Tetrax tetrax***. Dashed-lines represent 95% confidence intervals, grey dots represent the partial residual, and marks along the x-axis represent a single observation. Summary statistics of the GAM model is provided in Table S3.

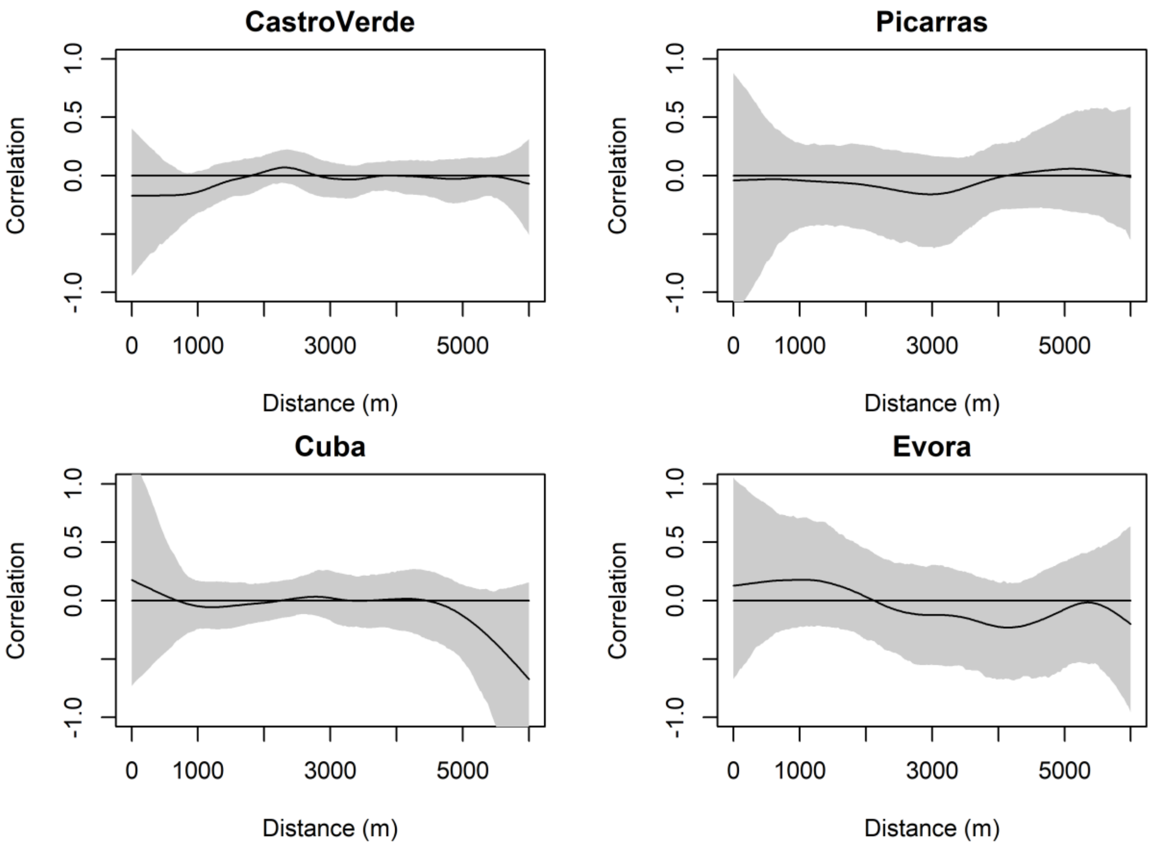

Figure S10 – Spline correlogram describing the spatial autocorrelation in the residuals of the **Bird Species Richness** GAM model. Lines represent the estimate (in the middle) and the 95% confidence envelopes (grey shadow) using 1000 bootstrap resamples.

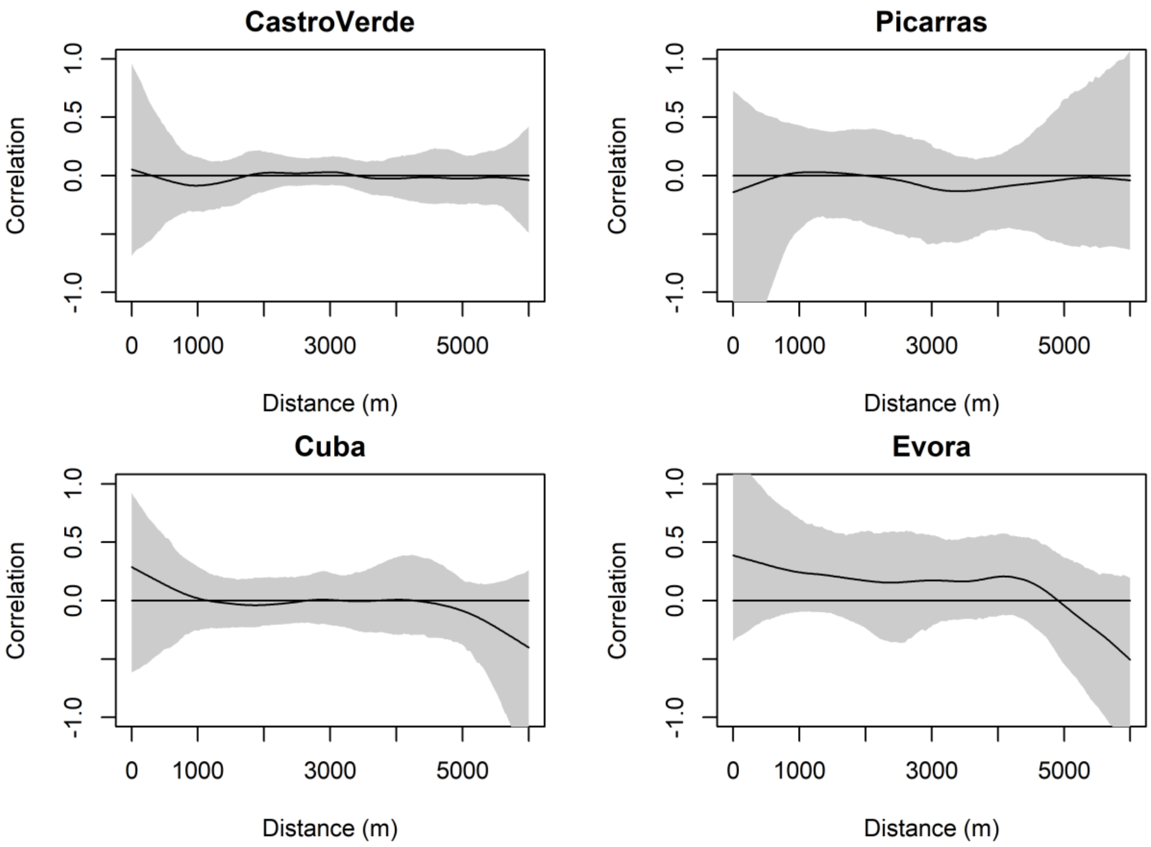

Figure S11 – Spline correlogram describing the spatial autocorrelation in the residuals of the **Grassland Bird Species Richness** GAM model. Lines represent the estimate (in the middle) and the 95% confidence envelopes (grey shadow) using 1000 bootstrap resamples.

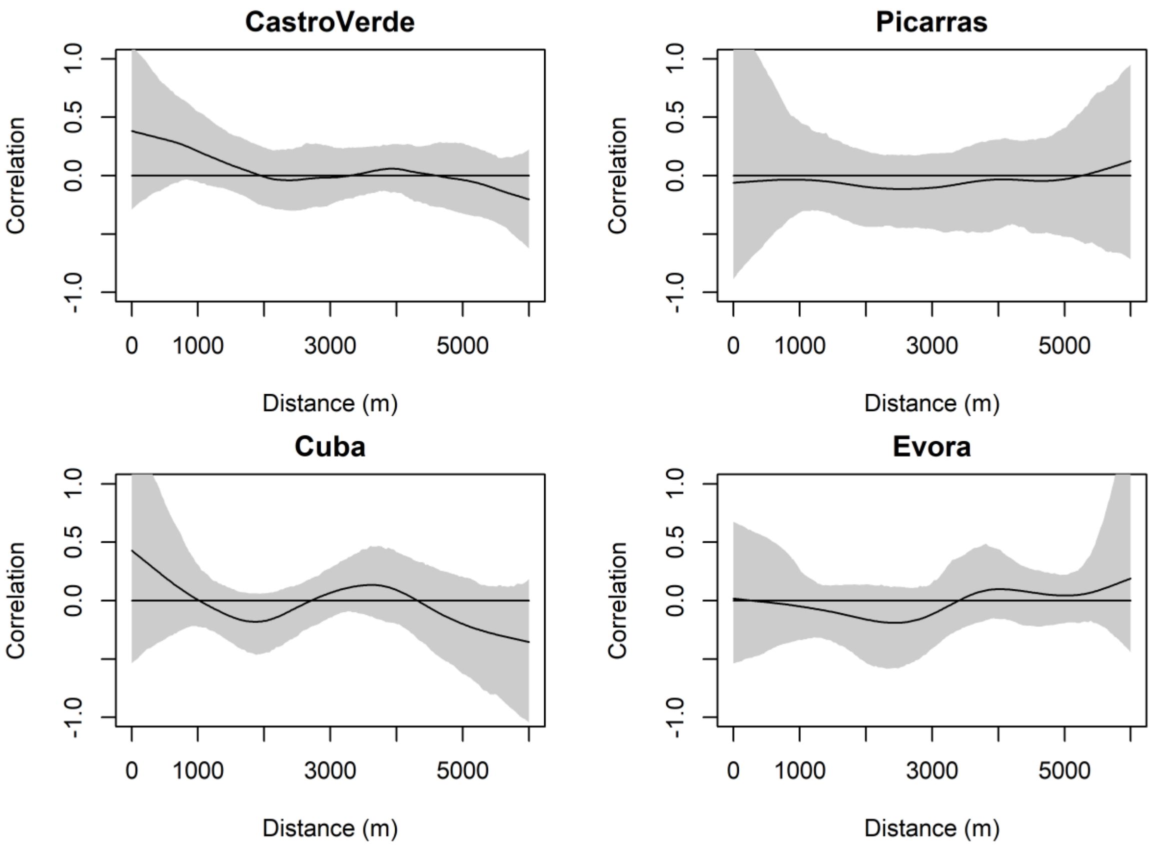

Figure S12 – Spline correlogram describing the spatial autocorrelation in the residuals of the **galerida larks *Galerida* spp.** GAM model. Lines represent the estimate (in the middle) and the 95% confidence envelopes (grey shadow) using 1000 bootstrap resamples.

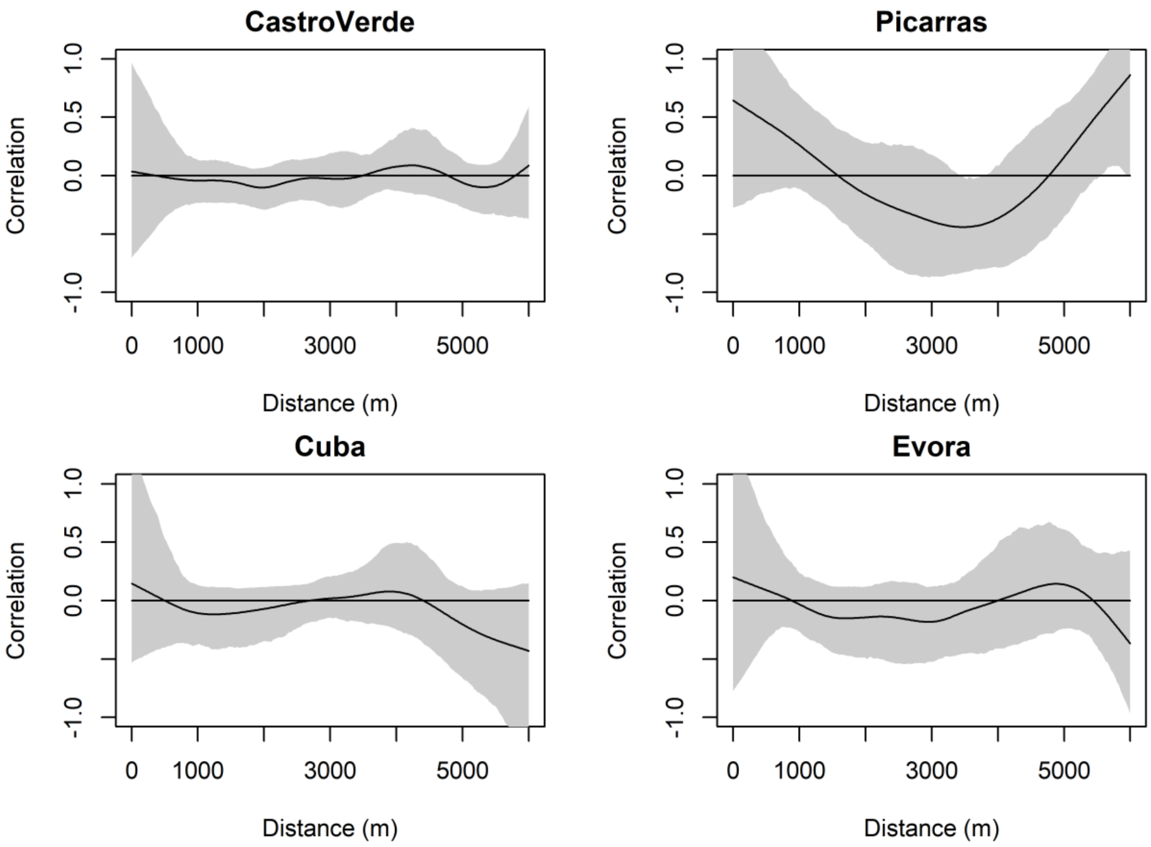

Figure S13 – Spline correlogram describing the spatial autocorrelation in the residuals of the **zitting cisticola *Cisticola juncidis*** GAM model. Lines represent the estimate (in the middle) and the 95% confidence envelopes (grey shadow) using 1000 bootstrap resamples.

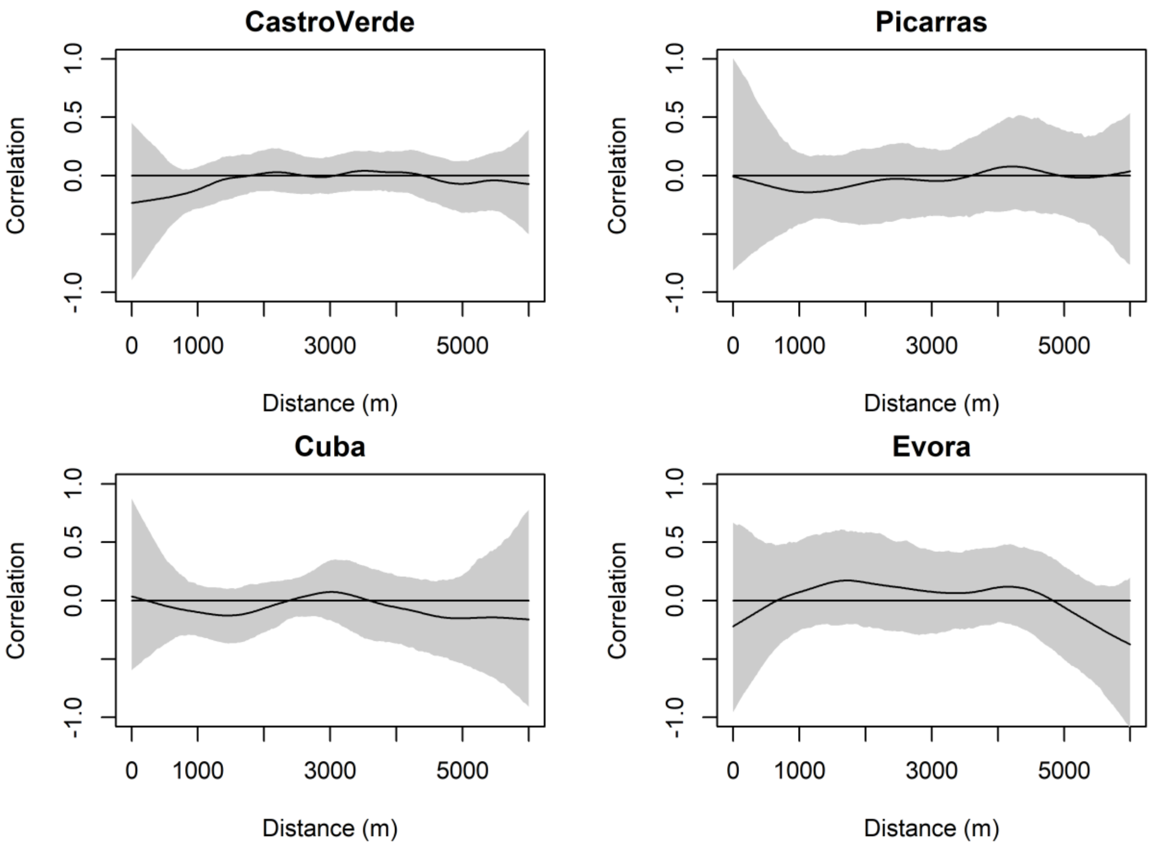

Figure S14 – Spline correlogram describing the spatial autocorrelation in the residuals of the **common quail *Coturnix coturnix*** GAM model. Lines represent the estimate (in the middle) and the 95% confidence envelopes (grey shadow) using 1000 bootstrap resamples.

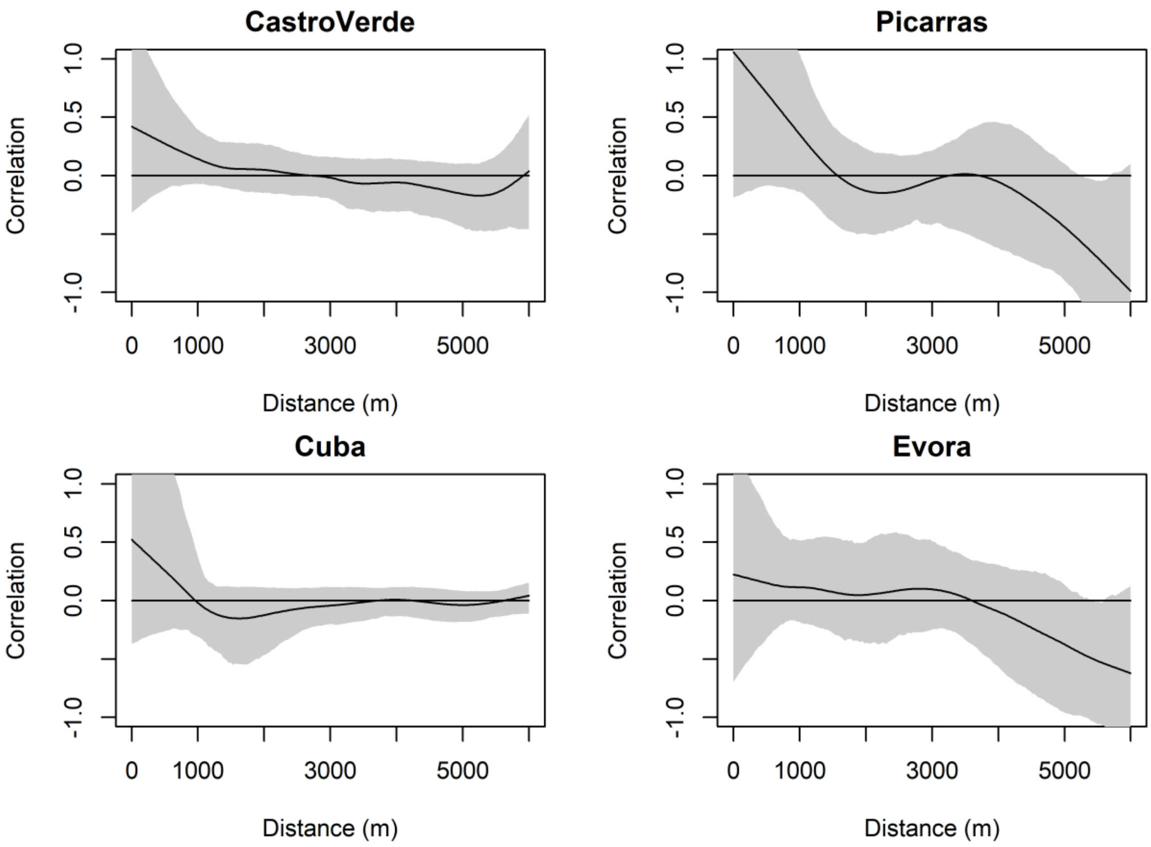

Figure S15 – Spline correlogram describing the spatial autocorrelation in the residuals of the **calandra lark *Melanocorypha calandra*** GAM model. Lines represent the estimate (in the middle) and the 95% confidence envelopes (grey shadow) using 1000 bootstrap resamples.

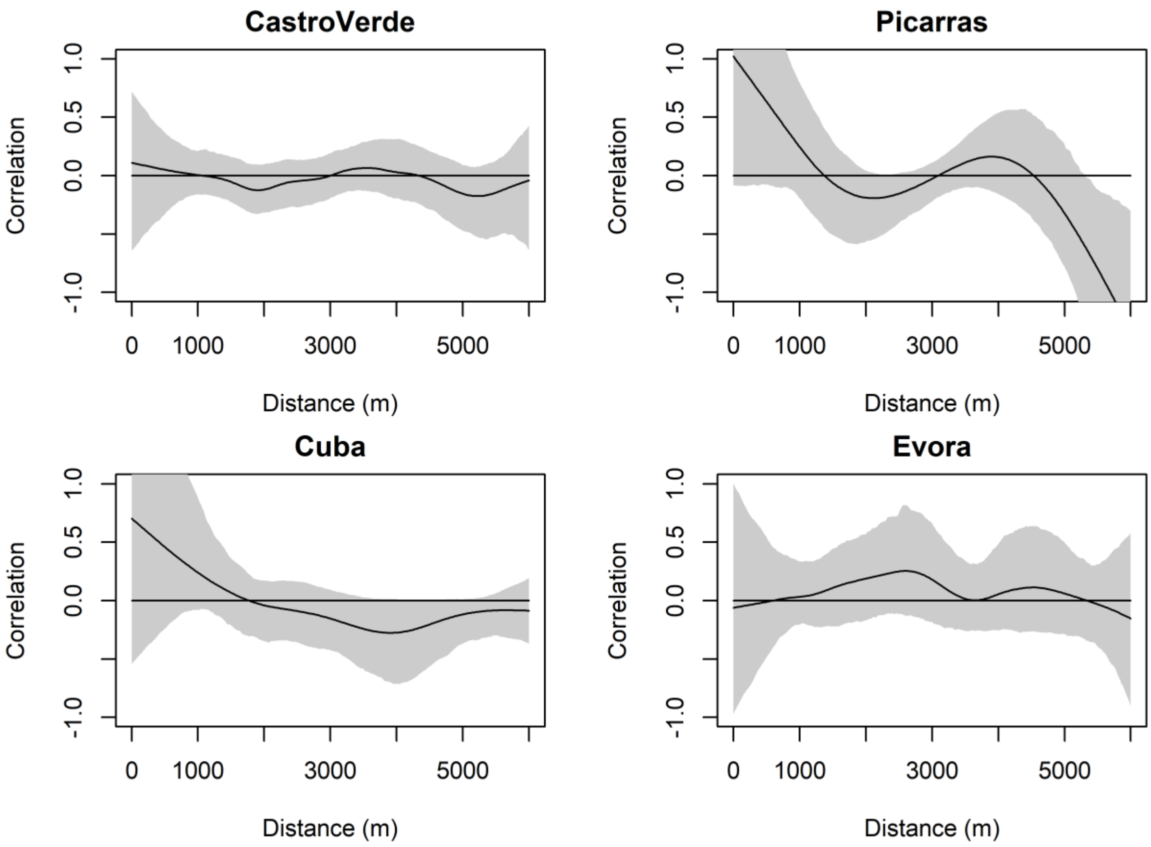

Figure S16 – Spline correlogram describing the spatial autocorrelation in the residuals of the **little bustard *Tetrax tetrax*** GAM model. Lines represent the estimate (in the middle) and the 95% confidence envelopes (grey shadow) using 1000 bootstrap resamples.

REFERENCES

BirdLife International (2021) European Red List of Birds. Luxembourg: Publications Office of the European Union.
